# Medical Items and News of Interest to the Physician

**Published:** 1885-07

**Authors:** 


### For the Use of Physicians.

CULLED FROM THE STANDARD MEDICAL JOURNALS OF THE WORLD.

NOTE.—These formulæ are intended only for the regular practitioner of medicine, and should be employed only by him, or under his immediate supervision.

**Corn Cure.**

Fld. ext. Cannabis indica, fl ʒ vj.

Salicylic acid, ʒ ivss.

Collodion, lb. ¼ av.

Mix.

—*Druggist Circular.*

**Catarrh Inhalant.**

Sulphuric ether 1½ ounces.

Chloroform 1 ounce.

Tinct. iodine ½ ounce.

Tinct, camphor ½ ounce.

Oil of tar ¼ ounce.

Mix and inhale, closing the nostril after each inhalation, and forcing the vapor into the nose. —*N. E. Medical Monthly.*

**Osgood's Cholagogue or Celebrated Ague Cure.**

R

Sulph. quinine 2 drms.
Fluid ext. leptandra 2 drms.
Saturated tinct. stillingia 4 oz.
Fluid ext. podophyllum 3 drms.
Oil of sassafras 10 drops.
Oil of wintergreen 10 drops.
New Orleans molasses. sufficient to make 8 ounces.

M. Dose one to two teaspoonfuls.—*Stearns' New Idea.*

**A Combination of Bromides for Epilepsy.**

ERLENMEYER (*Centralbl. f. Nervenheilk.; Centrrlbl. f. die ges. Ther.*) recommends the following formula;

Bromide of ammonium, 30 grains.
Bromide of potassium,
Bromide of sodium, aa ʒ.
Water, 24 ounces.

The whole to be given in the course of twenty-four hours.

It is said that the use of this mixture does not cause bromism, even acne not having been observed under its action, and that its control over the attacks is very decided.—*N. Y. Med. Jour.*

**Hair Tonic.**

Oil ricini. ℥ iij.

Spt. vini rect, ℥ xij.

Tr. cantharid, ʒ j.

Tr. Sang. can. ʒ ij.

M.

**Hemorrhoids.**

Potass. ʒ ij, ext. ergot liquid, ʒ iss' tr. nucis vomica, ʒ j. aromatic spts. of ammonia, ʒ iss, aqua, ℥ viij, is strongly recommended. All local applications are only palliative not curative. This is as good as any used.

**An Antiseptic Mouth Wash.**

MAGITOT (*Union Med.*) suggests the following:

Borate sodium, 15 grs,
Thymol, 8 grs.
Distilled water, 1 pint.

The mouth is to be washed several times daily, to correct fœtor of the breath. The solution also acts as a local anæsthetic in cases of decayed teeth.

**Tonic Cough Mixture.**

Dr. E. E. WILLIAMS, in a note to the *New England Medical Monthly*, states that he considers the following formula "unsurpassed";

Syrup of squill.
Camph. tr. of opium,
each 1 ℥.
Wine of antimony 1 ʒ.
Fl. ext. licorice,
Balsam copaiba each 3 ʒ,

Mix. Dose, a teaspoonful every two, three, or four hours, as the case requires.

**Kennkle's Vegetable Worm Syrup.**

UPON examination, we find that it is put up in an oval green bottle containing 4½ fluid ounces of an opaque, yellowish-brown, thin, syrupy liquid of slightly acid reaction. According to our examination, each bottle contains;

Santonin, 27 grains.
Oil sassafras, 1 minim.
Alcohol, 2 fl oz.
Fluid extract pink root, 2 fl oz.
Fluid extract dandelion, ½ fl oz.
Fluid extract golden seal, ¼ fl oz.
Molasses, ½ fl oz.

The santonin in a finely triturated condition.

A preparation of similar size of this character should be worth about $45 per gross or $4 per dozen.—*Stearns' New Idea:*

**"Common Colds."**

—As a useful prescription in "*common colds*," Prof. Bartholow offers the following :—

℞.
Codeiæ, gr. j
Syrup scillæ comp.,
Syrup tolu, aa f ℥ ss. M.
SIG.—A teaspoonful pro re nata.

---

**Hæmoptysis.**

—For a young woman, æt. 30, suffering from *hœmoptysis*, consequent upon incipient phthisis, Prof. Da Costa gave—

℞.

Acidi sulphurici dilut., gtt. v

Morphinæ sulphat., gr. $\frac{1}{48}$

Elixir. simplicis. f ℥ ss. M.

SIG.—To be taken as required.

She was also directed to take arseniate of sodium, gr. $\frac{1}{40}$, ter die, after meals.

---

**Toothache Cure**

A. GAUDET makes a report on a preparation which has come under his possession of a female customer, who praised it greatly. He succeeded, after numerous trials, in ascertaining its composition, which is as follows :

Mastic, tears 8 parts.
Balsam Peru, 5 parts.
Chloroform, 14 parts.

Dissolve the mastic in the chloroform, then add the balsam. After twelve or fifteen hours, filter.

For use, place two or three drops upon a small pellet of cotton, which is to be introduced into the cavity of the hollow tooth.—*L'Union Pharm.*

---

**Eye Waters.**

P- W. (Providence, R. I).—The following are found in Dick's Encyclopædia, p,503 :

ASTRINGENT EYE WATER.

Sulphate of zinc 20 grains.
Distilled water ½ pint.
Dissolve.

For chronic and ordinary opththalmia, and for weak, lax, watery, and irritable eyes. If there be much pain, five or six grains acetate of morphia or two fluid drachms of wine of opium may be added.

ANOTHER EYE WATER.

Solution acetate of ammonia, 2 fl ounces.
Distilled water, hot 6 fl ounces.
Soft extract of opium, 10 grains.

—*Druggist Circular.*

**Ticdouloureux.**

*Epileptiform tic douloureux*, dependent, as was thought, upon an exostosis.—

℞.

Potassii iodidi, ℈ j

Sodii bromidi, ℈ ij.

M.

SG.—Bis die.

---

**Cholera Infantum.**

℞

Tinct opii, gtt, xvj.

Spts. ammon aromat. ʒ ss-j.

Bismuth subnitrat ʒ ij.

Syr. simplic ʒ ss.

Mistur cretæ ℥ iss.

M. Sig.—One teaspoonful every two or three hours to a child of eight to twelve months, until vomiting and diarrhœa are controlled.—*Dr. J. Lewis Smith in N. E. Med. Monthly.*

---

**Mixture for Plastic.**

THE following mixture is recommended by Dr. Brocke (*Monatsh. f. Prakt. Derm.*, 1885 21) as in many instances preferable to gelatin mixtures :

Powdered talc 2 parts.
Wheat flour 1 part.
White wax 3 parts.

Mix the powders, melt the wax, and, when not too hot, make a thorough mixture of all the ingredients. The mass may be colored if desired.—*Pharm. Centralh.*

---

**Perspiration of the Feet.**

*F. E. E.* (*Ottumwa, Iowa*) asks for something that will toughen the feet and prevent them from perspiring. In our issue for March, 1884, the following directions were given for the treatment of perspiring feet ; Apply with a sponge without rubbing, a solution of 30 grains each of burnt alum and boric acid and 1 ounce of rose water, just as soon as the shoes and stockings are removed. Repeat every two or three days in the evening. A pharmacist, who regards the difficulty as due to fermentation caused by the paste used by shoemakers for fastening insoles into shoes, directs a powder consisting of about equal parts of boric and salicylic acids to be sprinkled in the shoes, and, we are told, the trouble ceases. Another pharmacist, who has devoted considerable attention to the difficulty in question, highly recommends the application of a mixture of 1 part of powdered oleate of zinc with ten parts of starch.—*Ex.*

**Hypodermic Solution.**

We clip the following very useful formulae from the *College and Clinical Record:*

—Prof. Bartholow recommends the following as a non-irritating preparation of *quinia for hypodermatic use:*—

℞

Quininæ hydrobromat., gr. xlviij,

Aquæ destillat, f ℥ ss. M.

Dissolve, and by heat if necessary.

SIG.—Twenty minims contain four grains.

---

**Anti-Hemorrhagic.**

—As an *anti-hemorrhagic* combination, Prof. Bartholow speaks highly of—

℞

Extract ipecac fluid. f ʒ ij

Extract ergotæ fluid., f ʒ iv

Extract digitalis fluid., f ʒ ij M.

SIG.—Thirty minims to a drachm at a dose, pro re nata.

---

**Chronic Rheumatism.**

—For *chronic rheumatism* in an anæmic woman, in whom the joints of both hands were stiff and swollen, together with the large joints of the body, Dr. J. C. Wilson, of the medical clinic, prescribed—Ol. morrhuæ, ℥ ss, bis die; flannels to be worn next the skin, and—

℞ Mist. ferri et ammonii acetat., f ℥ ss.

Strychninæ sulphat, gr. $\frac{1}{48}$ M.

SIG.—To be taken ter die, after meals.

---

**Epistaxis.**

—Prof. Bartholow, for a case of frequent epistaxis, occurring in a young man of twenty-five years of age, recommended the following prescription, to maintain the tonicity of the blood:—

℞

Ergotæ (aq. ext.), gr. ij
Ferri sulphat., gr. j
Extract. nucis vomicæ, gr. $\frac{1}{4}$. M.

SIG.—In pill, ter die.

---

**Constipation and Dyspepsia.**

—Prof. Da Costa prescribed, for a woman complaining of *constipation* and *dyspepsia*, the following:—

℞

Ext. belladonnæ, gr. $\frac{1}{16}$

Strychninæ sulphat., gr. $\frac{1}{60}$

Pulv. aloes, gr. $\frac{1}{10}$

Olei cajuputi, gtt. j

Extract gentianæ, q. s.

SIG.—One pill ter die.

**Mitral Murmur.**

—Dr. O. P. Rex, Physician to the Hospital, prescribed for a man with a *mitral regurgitant murmur*, the following:—

℞

Pulv. digitalis, gr. $\frac{1}{8}$

Extract belladonnæ, gr. $\frac{1}{12}$

Extract gentianæ, q. s. M.

SIG.—In pill, ter die.

---

**Gastric Vertigo.**

—For *gastric vertigo*, with disturbed circulation, Prof. Da Costa administered, in the case of a man—

℞

Argenti oxidi gr. ss
Ext. hyoscyami, gr. ij M.

SIG.—In pill form ter die.

He also prescribed pepsin at meals.

---

**Diarrhoea.**

—For the *diarrhœa* often attendant upon puerperal septicæmia, Prof. Parvin recommends the following:

℞

Ol. ricini, ʒ j

Tinct. opii,

Ol. terebinthinæ. aa gtt. v. M.

SIG.—Take pro re nata.

---

**Chorea.**

—In the case of a young man, aged 21, suffering from a well-marked attack of *chorea*, that could not be linked to any history of rheumatism, but possessing, as did his father, a most irritable temperament, to which Dr. Rex attributed the cause of the trouble, he prescribed—

℞

Potassii iodidi, gr. vij

Extract cimicifugæ fluid., gtt. xx

Syrup sarsap. comp.,

Aquæ, aa f ʒ j M.

SIG.—To be taken ter die.

**Fothergill's Formula for Asthma.**

THE following is Dr. Fothergill's formula for asthma (*Med. Sum.*):

℞

Tinct. lobeliæ ℥ v.

Ammonii iodidi ʒ ij.

Ammonii bromidi ʒ iij.

Syr. tolu ℥ iij,

M. Teaspoonful every one, two, three, or four hours. This gives relief in a few minutes, and sometimes the relief is permanent.

**Treatment of Bunions.**

Tincture of iodine applied locally, will, in my opinion, afford better results than any other remedy. I have seen both toe joints so much out of shape that boots had to be made especially for the feet. I have used the tincture of iodine and materially reduced these joints to almost normal size. It is the best remedy I ever tried.—*Ex.*

**Treatment of Ephelis or Freckles.**

Dr. Hebra recommends :

R.

Mercury bichloride. 1 part,

Cologne, 99 parts,

As a local application night and morning.

Preparations of arsenic should not be used in simple skin diseases.

*Med. Summary.*

**Gonorrhoea.**

According to Prof. Bartholow, the following in gonorrhœa, after the acute stage is efficacious:

R.

Zinci sulphat.,

Plumbi acetat, aa grs. viij,

Ammonii chlorid.,

Aluminis, aa grs. iv,

Aqua rosæ, f ℥ j.

SIG.—As an injection.—*Ex.*

**Amenorrhoea.**

For amenorrhœa dependent upon debility, Prof. Parvin advises the following, speaking very highly of its utility:

R.

Ferri sulphat exsiccat,

Aloes, aa gr. j,

Olei terebinthinæ, m j.

Ft. pil.

M.

SIG.—One ter die.

**Anemia.**

A favorite prescription of Dr. Da Costa in marked idiopathic anemia is ;

R.

Ferri sulph,

Potassii carb., aa ʒ j.

Ft. pil. No. xl.

SIG.—One after meals for first week; increase dose in second week, etc.

If the patient is a female, suspend treatment during menstruation.—*Ex.*

**Dipsomania.**

Aromatic spts. ammonia, tr. valerian and spts. lavandulæ in ʒ j doses, every two hours, gives good and satisfactory results.

**Habitual constipation (Prof. Bortholow):—**

R.

Resinæ podophylli, gr. vj

Ext. belladonnæ,

Ext. physostigmatis. aa gr. iij

Ft. pil., No. xij.

M.

SIG.—One pill each night.

—*Ex.*

**Treatment of Furuncle Boil.**

If seen before the formation of pus the boil may be touched with a stick of argentic nitrate and absorbed. The pain and stinging sensation caused by it can be released by the local application of the tincture of opium. After pus has been formed, exudation of it must be encouraged by warm applications, and the knife used. The aspirator will also answer to remove the thinner and more watery portion of the boil. Previous to evacuating the furuncle, the bowels must be freely emptied of their contents and tonics given to promote digestion. Exercise, too, is necessary. Boils are frequently called abscesses and treated as such, the treatment being similar. These painful affections arise from too *much* or too *little* blood. If from anemia, iron and quinine, if from plethora or too much blood, exercise, cathartics and diet of a low order.—*Med. Summary.*

**Paper Towels for Surgical Purposes.**

Dr. ROBERTS, of Philadelphia, has been using with much satisfaction Japanese paper handkerchiefs for drying wounds. Sponges are so seldom and with such difficulty perfectly cleansed after being once used, that they are never employed in the clinic. Ordinary cotton or linen towels are much preferable to sponges, but, if dirty, are liable to introduce septic material into wounds. The paper towels answer the same purpose as cotton ones, and are so cheap that they can be thrown away after being used. They cost from $6 to $7.50 a thousand. The cost of washing a large number of ordinary towels is thus avoided. The paper towels are scarcely suitable for drying hands, after washing unless several towels are used at once, because the large amount of moisture on the hands soon saturates a single towel. For removing blood from wounds, a paper towel is crumpled up into a sort of a ball, and then used as a sponge. Such balls absorb blood rapidly. The crude ornamental pictures in color, on the towels are of no advantage nor are they as far as known any objection.—*Gaillard's Med Jour.*

**Elimination of Phosphoric Acid in the Urine in Insanity and Epilepsy.**

A. LAILLER'S (*Comp. Rend.*, 99, 572,), obtained from many hundred analyses made while resident officer in a large asylum, agree with those of Mariet. In acute delirium, phosphoric acid and urea are eliminated in notable excess ; in excitable mania the phosphoric acid is in slight excess, whilst the amount of urea is normal ; and in simple insanity the urine has the normal composition. In acute or excitable lypemania, the amount of urea eliminated is abnormally high, whilst that of phosphoric acid is abnormally low. In simple lypemania the composition of the urine is normal.

In general paralysis the elimination of both phosphoric acid and urea is related to the general morbid conditions of the patient. At, or immediately after, epileptic seizures, the urine contains a high portion of phosphoric acid and a low portion of urea. If the seizures succeed one another rapidly, the proportion of both phosphoric acid and urea is increased ; in the interval between seizures the urine has the normal composition.—*Journal Chem. Soc.*

(TO THE BISTOURY.)

**Nervous Headache.**

Spirit of ether in 30 minum doses gives instant relief and rarely fails to cure the affection.

(FOR THE BISTOURY.)

**Acute Rheumatism.**

℞

Sodii bicarb.

Potassii acetatis, aa ʒ iss.

Tinct cinchonæ, ʒ iss.

Decocti cinchonæ flav., ℥ iss.

M.—For one dose.

(FOR THE BISTOURY.)

**Lithiasis.**

℞
Potassii bicarbonatis, ʒ xii.
Acidi citrici, gr. viii, to xxiv.
Aquam, ad. f ℥ xii.

One or two tablespoonfuls in a glass of water thrice daily.

(FOR THE BISTOURY.)

**Asthma.**

Nitrite of amyl is a remedy of the greatest utility in asthma when purely spasmodic. Inhilation of several drops relaxes the spasm immediately. Some care should be used in not prescribing it when cardiac disease is present.

[I have experienced the best results with nitrite of amyl in the affection spoken of.—ED.]

(FOR THE BISTOURY.)

**Diabetes.**

Since ergot was first employed and recommended by Prof. J. M. Dacosta in diabetes insipidus many physicians have tried it and cases have been reported cured.

The remedy must be pushed perseveringly and given in full doses.

(FOR THE BISTOURY.)

**Pneumonia.**

Prof. A. B. Palmer when called to a patient within twenty-four hours of the chill, he immediately gives:

℞

Morphiæ sulphatis, gr. $\frac{1}{4}$—$\frac{1}{2}$.

Quiniæ sulphatis, gr. vi—x.

M.—In about an hour this induces free perspiration.

(FOR THE BISTOURY.)

**Acute Bronchitis.**

Prof. J. M. Dacosta prescribes:

℞
Vini ipecacuanhae, ʒ ij.
Liquor potassii citratis, ℥ iv.
Tinct. opii. camph.
Syrup. acaciae, aa ℥ j.

A tablespoonful thrice daily in the first stage of ordinary bronchitis.

(FOR THE BISTOURY.)

**Spermatorrhœa.**

The following has been prescribed at the Jefferson hospital by Prof. Bartholow for this trouble, especially in the spermatorrhœa of plethora:

Infusion digitalis, ℥ viii.

Potassii bromidi, ℥ i.—M.

A tablespoonful morning and night, for a week, then at night only.

(FOR THE BISTOURY.)

**Belladonna Poisoning.**

I have had frequently cases come to me complaining of dryness of the mucous membrane of the mouth and lips, severe headache with vertigo, flushing of the face and often with a nauseating feature. The patient cannot give any satisfacfactory explanation of the cause, but upon questioning I find all of the symptoms caused by wearing a belladonna plaster.

The people in general too often resort to home treatment without knowing otherwise than they hear it is "good for the complaint."

